# A microRNA biomarker of hepatocellular carcinoma recurrence following liver transplantation accounting for within-patient heterogeneity

**DOI:** 10.1186/s12920-016-0179-4

**Published:** 2016-04-08

**Authors:** Qing Yan Xie, Anthony Almudevar, Christa L. Whitney-Miller, Christopher T. Barry, Matthew N. McCall

**Affiliations:** Department of Biostatistics and Computational Biology, University of Rochester Medical Center, Rochester, NY USA; Department of Pathology, University of Rochester Medical Center, Rochester, NY USA; Department of Surgery, University of Rochester Medical Center, Rochester, NY USA; Department of Biomedical Genetics, University of Rochester Medical Center, Rochester, NY USA

**Keywords:** microRNA, Biomarker, Hepatocellular carcinoma (HCC), Liver transplantation, Recurrence

## Abstract

**Background:**

Liver cancer, of which hepatocellular carcinoma (HCC) is by far the most common type, is the second most deadly cancer (746,000 deaths in 2012). Currently, the only curative treatment for HCC is surgery to remove the malignancy (resection) or to remove the entire diseased liver followed by transplantation of healthy liver tissue. Given the shortage of healthy livers, it is crucial to provide transplants to patients that have the best chance of long-term survival. Currently, transplantation is determined via the Milan criteria—patients within Milan (single tumor < 5 cm or 2–3 tumors < 3 cm with no extrahepatic spread nor intrahepatic vascular invasion) are typically eligible for transplantation. However, combining microRNA expression profiling with the Milan criteria can improve prediction of recurrence.

HCC often presents with multiple distinct tumor foci arising from local spread of a primary tumor or from the oncogenic predisposition of the diseased liver. Substantial genomic heterogeneity between tumor foci within a single patient has been reported; therefore, biomarker development must account for the possibility of highly heterogeneous genomic profiles from the same individual.

**Methods:**

MicroRNA profiling was performed on 180 HCC tumor samples from 89 patients who underwent liver transplantation at the University of Rochester Medical Center. The primary outcome was recurrence-free survival time, and patients were observed for 3 years post-transplantation.

**Results:**

MicroRNA expression profiles were used to develop a biomarker that distinguishes HCC patients at greater risk of recurrence post-transplantation. Unsupervised clustering uncovered two distinct subgroups with vast differences in standard transplantation selection criteria and recurrence-free survival times. These subgroups were subsequently used to identify microRNAs strongly associated with HCC recurrence. Our results show that reduced expression of five specific microRNAs is significantly associated with HCC recurrence post-transplantation.

**Conclusions:**

MicroRNA profiling of distinct tumor foci, coupled with methods that address within-subject tumor heterogeneity, has the potential to significantly improve prediction of HCC recurrence post-transplantation. The development of a clinically applicable HCC biomarker would inform treatment options for patients and contribute to liver transplant selection criteria for practitioners.

**Electronic supplementary material:**

The online version of this article (doi:10.1186/s12920-016-0179-4) contains supplementary material, which is available to authorized users.

## Background

Hepatocellular carcinoma (HCC) is one of the most common malignancies worldwide, accounting for the second most cancer-related deaths [1, 2]. In the U.S., it has been predicted by 2030 to become the third leading cause of cancer-related death, surpassing breast, prostate, and colorectal cancers [3]. The only curative treatment is surgery: either tumor resection or liver transplantation. However, patients undergoing these treatments still have a high risk of recurrence. Both resection and transplantation result in 80 % 5-year patient survival rates in appropriately selected patients. However, the recurrence rate in 5 years for resection is 70 % whereas with transplantation the 5-year recurrence rate is 15–20 % [4]. Even though the 5-year HCC recurrence rate after transplantation seems acceptably low at 15–20 %, improved selection criteria would further optimize outcomes and therefore more efficiently use the precious resource of donor liver tissue. The current selection criteria for transplantation, the Milan criteria, are a single tumor < 5 cm or 2–3 tumors < 3 cm with no extrahepatic spread or intrahepatic vascular invasion [5]. While the Milan criteria alone perform reasonably well, combining the Milan criteria with a microRNA biomarker has been shown to improve prediction of recurrence [6].

MicroRNAs are receiving growing attention as biomarkers due to their diverse role in cellular regulation. In cancer, microRNAs have shown promise as both diagnostic and prognostic biomarkers [7]. A recent study reported a microRNA biomarker of HCC recurrence after liver transplantation from serum exosome samples [8]. Other studies have proposed microRNA biomarkers of HCC recurrence based on microRNA expression profiles from solid tumor biopsies [9–12].

Tumor biopsy followed by histopathology, or more recently genomic analysis, is a standard procedure to assess the type, severity, and prognosis of many cancers. Typically only one biopsy is taken from each patient. While this may be sufficient to determine whether a mass is malignant or benign, it is insufficient to capture within-patient tumor heterogeneity, which has been shown to exist both between tumor foci [13, 14] and within a single tumor [15, 16]. Therefore, biopsying and analyzing only one sample per patient runs the risk of failing to capture the cells driving the malignant phenotype. While the goal of precision medicine is to harness between-patient tumor heterogeneity to tailor treatment to specific features of an individual's cancer profile, within-patient tumor heterogeneity poses a serious challenge to this goal. Within-patient tumor heterogeneity affects both biomarker development and application. During development, heterogeneity will reduce the power to detect genomic signatures associated with the phenotype of interest. Even if a biomarker is successfully developed, both good and poor prognosis signatures may be present within the same patient complicating clinical application.

Hepatocellular carcinoma (HCC) is particularly well suited to the study of within-patient heterogeneity because it often presents with multiple tumor foci. In patients with multifocal HCC, the individual lesions can arise from either local dissemination of the primary tumor or from the oncogenic predisposition of the diseased liver. In the latter case, a patient with multifocal HCC may have multiple tumors that are clonally unrelated and presumably exhibit distinct genomic profiles. This presents a challenge to genomic analyses attempting to associate sample-level genomic profiles (e.g. microRNA expression) with patient-level phenotypic data (e.g. recurrence-free survival). Furthermore, recurrence of HCC post-transplantation is commonly associated with multifocal tumors.

The goals of this study are to: (1) further examine the association between microRNA expression, current transplantation selection criteria, and HCC recurrence, and (2) to develop a biomarker of HCC recurrence post-transplantation that is able to incorporate information from multiple tumor foci. The approach proposed in this paper addresses the challenge of within-patient heterogeneity by developing a sample-level model of recurrence and coupling this model with patient-level information to make predictions.

## Methods

### Patient and sample description

The data are comprised of 180 tumor samples from 89 HCC patients who underwent liver transplantation at the University of Rochester Medical Center (GEO Series accession number GSE67140). This study was performed with approval of the University of Rochester Research Subjects Review Board (RSRB00029467). Liver explant pathology specimens (paraffin embedded blocks) from patients undergoing liver transplant for HCC were de-identified prior to processing and analysis, so individuals were exempt from consent. Demographics of the patient cohort are shown in Additional file 1: Table S1. Of these 97 tumor samples from 69 HCC patients were previously described in Barry et al. [6]. By nearly doubling the number of tumor samples, we are able to investigate the effect of within-subject heterogeneity on microRNA biomarkers of HCC recurrence. Each patient was observed for 3 years and recurrence-free survival time (or censoring time) was recorded.

### MicroRNA purification and array hybridization

Samples were isolated, hybridized, and processed exactly as in Barry et al. [6]. The Roche High Pure miRNA isolation kit (Roche Diagnostics, Mannheim, Germany) was used to isolate miRNA from formalin-fixed paraffin embedded (FFPE) liver tumor tissues. Samples were assessed for the presence of enriched miRNA using an Experion Bioanalyzer (Bio-Rad, Hercules, CA, USA). MicroRNA labeling was performed using the FlashTag Biotin RNA labeling kit (Genisphere, Hatfield, PA, USA). MicroRNA expression was assessed using Affymetrix GeneChip miRNA 1.0 microarrays (Affymetrix, Santa Clara, CA, USA). Array hybridization, washing, and staining was performed at the Upstate Medical University microarray core facility in Syracuse, NY, according to the manufacturer’s instructions. Arrays were scanned using a GeneChip Scanner 7G Plus.

### Quality control and data preprocessing

Array quality was assessed by visual inspection of residual pseudo chip images, Normalized Unscaled Standard Error (NUSE) medians and interquartile ranges, and Relative Log Expression (RLE) medians and interquartile ranges [17]. Of the 10 poor quality arrays, 6 were rehybridized resulting in improved quality. There was insufficient genetic material to rehybridize the other four poor quality samples. Analysis was performed on 176 samples of acceptable quality from 89 HCC patients.

The data included in this study span four distinct batches, based on the date on which the microarray hybridization occurred. Samples from the same patient sometimes span multiple batches. All samples were processed and analyzed in the same manner.

Arrays were preprocessed using Robust Multi-array Average (RMA) [18]. Subsequent analysis was restricted to the 847 human microRNA probe sets. Recurrence-free survival time was the primary outcome of interest in these data. All data analyses were performed using the R/Bioconductor statistical computing environment [19]. The processed data and R scripts needed to reproduce all analyses were submitted with this manuscript as additional data files and made freely available on GitHub: https://github.com/mccallm/HCCmicroRNA.

### MicroRNA expression in multifocal tissue samples

For patients with unifocal HCC, patient-level and sample-level models are identical. In other words, recurrence post-transplantation is predicted based on the single observed sample. In the case of multifocal HCC, we obtained multiple samples from distinct tumor foci. In this case, it is important to distinguish between patient-level and sample-level modeling. Samples from the same individual may have vastly different genomic profiles. As such, it is crucial to distinguish between the sample(s) that are driving recurrence and those that are not.

### Visualization and examination of sample-level clustering

First, we used the t-Distributed Stochastic Neighbor Embedding (t-SNE) technique to visualize the distribution of samples. The t-SNE is a nonlinear dimensionality reduction technique that facilitates visualization of high dimensional data in two or three dimensions [20]. It is implemented in the R package Rtsne. Principal Component Analysis (PCA) was also used to visualize the data in a low dimensional space.

Second, we use several unsupervised learning algorithms to identify potential subgroups within the data. We evaluated nine different unsupervised learning methods (Hierarchical clustering, KMeans, DiANA, Fanny, Pam, Clara, Som, Sota and Model based clustering) using the clValid package, and found that KMeans with 2 clusters resulted in the clearest separation between subgroups in our data. This appears consistent with the results from the t-SNE analysis.

### Feature selection

One KMeans cluster (cluster one) consists mostly of samples from HCC recurrent patients, and the other cluster (cluster two) includes samples from both recurrent and non-recurrent patients. Samples from recurrent patients in cluster one are labeled as *poor prognosis,* and samples from non-recurrent patients in cluster two are labeled as *good prognosis*. The former class is comprised of 22 samples, and the latter contains 66 samples. These class labels address the possible ambiguity for multifocal patients with samples in both clusters and are used as training data for feature selection. By selecting the training data in this manner, we guarantee that there are no patients who have samples in both the training and testing sets.

We used the 88 selected samples and their class labels to determine features that are associated with poor prognosis. Mutual information was used to measure the contribution of each feature to sample classification. The mutual information of a feature (X) and a class (Y) is the expected value of the point-wise mutual information over the HCC recurrence and non-recurrence outcomes, as follows:

$$ I\left(X;Y\right)={\displaystyle \sum_{y\in Y}{\displaystyle \sum_{x\in X}p\left(x,y\right) \log \left(\frac{p\left(x,y\right)}{p(x)p(y)}\right)},} $$

We do not assume a linear association between microRNA expression and HCC recurrence. Instead we discretize microRNA expression into four intervals: [Min,Q1], [Q1,Q2], [Q2,Q3], [Q3,Max] where Q1, Q2, and Q3 represent the first, second, and third quartiles of expression for a given feature. Mutual information was calculated for each feature across the four intervals. The greater the mutual information value, the more the feature is associated with HCC recurrence.

### Biomarker assessment

We applied Naïve Bayes and Support Vector Machine learning models to assess the ability of the proposed biomarker to predict sample membership in the subgroups. The Naive Bayes and Support Vector Machine functions are from R package, e1071 version 1.6-4. The Support Vector Machine kernel type used was a radial basis with a cost value of 1000. For both classifiers, the 88 previously described samples were used as training data and the remaining 88 samples as test data. While multiple samples may come from the same patient, no patient had samples in both the training and test data sets.

## Results

### Visualization of microRNA Expression Reveals Two Subgroups

**Fig. 1:**
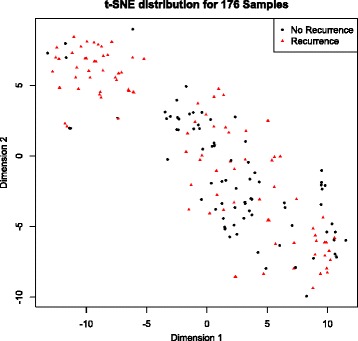
Two-dimensional representation of microRNA expression profiles. 176 samples with 847 dimensions are mapped into two dimensions via t-Dimensional Stochastic Neighbor Embedding using the Rtsne package. Samples closer together have more similar microRNA expression profiles. The samples from HCC recurrent patients are indicated with red triangles. The samples from non-recurrent patients are indicated with black circles. The majority of samples in the upper left are from recurrent patients; those in the bottom right are from a mixture of recurrent and non-recurrent patients

The data consist of 847 microRNAs measured across 176 samples. t-Distributed Stochastic Neighbor Embedding (t-SNE) facilitates visualization of high dimensional data in a low-dimensional space [20]. Samples that cluster in the low-dimensional space have a higher probability of association in higher dimensions. Projection of the microRNA expression data into two-dimensional space (Fig. 1) shows that the samples form two distinct subgroups. In the top left subgroup, most of the samples are from recurrent patients, while samples in the bottom right subgroup are from a mix of recurrent and non-recurrent patients. A plot of the first two principal components (Additional file 1: Figure S1) also shows separation into two distinct subgroups. Because recurrence applies to patients not samples, unsupervised sample-level analyses have the potential to uncover within-patient heterogeneity.

### Unsupervised Clustering into Two Subgroups

**Fig. 2:**
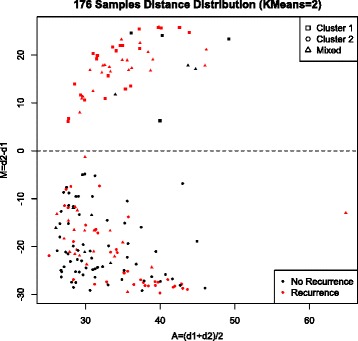
MA-plot of distance between the microRNA expression profile of each sample and the average expression profile of each subgroup. Large values of M correspond to samples that are far more similar to the average expression profile of subgroup 1 than subgroup 2. Large values of A correspond to samples that differ from the average expression profile of both subgroups. The dashed horizontal line (y = 0) indicates samples that are equidistant from both cluster centers. A square indicates a sample from a patient all of whose samples are from cluster 1. A circle indicates a sample from a patient all of whose samples are from cluster 2. A triangle indicates a sample from a patient whose samples are from both cluster 1 and cluster 2

To gain more insight into the groups observed in Fig. 1, we used KMeans clustering to group the data into two clusters. Similar to the t-SNE grouping, cluster 1 was comprised primarily of samples from recurrent patients, and cluster 2 was comprised of samples from both recurrent and non-recurrent patients. For each sample, we calculated the distance between its microRNA expression profile and the average expression profile of each subgroup (i.e. the two KMeans cluster centers). An MA-plot of the difference in distances (M = d2-d1) versus the average distance (A = {d1 + d2}/2) shows a clear separation between the two subgroups (Fig. 2). Subgroup 1 (above the dashed line in Fig. 2) is comprised of 50 samples, 43 of which are from recurrent patients and 7 of which are from non-recurrent patients. Subgroup 2 (below the dashed line in Fig. 2) is comprised of 126 samples, 71 of which are from non-recurrent patients and 55 of which are from recurrent patients. The data were acquired in four batches as described in the Methods Section. The batch in which the data were collected and analyzed does not appear to be strongly associated with subgroup, with the possible exception of batch 3, which is comprised of primarily non-recurrent patients (Additional file 1: Figure S2).

In Fig. 2, the labels, *Recurrence* and *No Recurrence,* are applied to all samples from a given patient; however, not all of the samples from that patient necessarily contributed to the recurrence. Since a patient with multifocal disease may have multiple samples analyzed, and just one sample might be responsible for the recurrence, the samples in cluster 2 could include relatively benign samples from either HCC recurrent patients or non-recurrent patients. In fact, 21 out of 55 samples (38 %) from recurrent patients in cluster 2 come from patients with at least one sample in cluster 1. In contrast, only 5 out of 71 samples (7 %) from non-recurrent patients in cluster 2 come from patients with at least one sample in cluster 1. This suggests that many of the samples from recurrent patients that fall in cluster 2 may not be responsible for the recurrence; rather a different sample from the same patient that falls in cluster 1 may be responsible for the recurrence. This highlights the rationale for using unsupervised clustering – the relationship between recurrence status and microRNA expression is complicated by the heterogeneity between samples from the same patient.

### Sample-Level Clustering Is Associated with Patient Survival

**Fig. 3:**
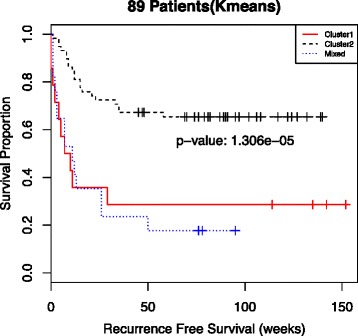
Kaplan-Meier curves of recurrence-free survival for all 89 patients stratified by subgroup membership of individual samples. The *p*-value suggests that recurrence-free survival time is strongly differentiated by subgroup. The red curve includes 14 patients with samples from cluster 1 only. The black dashed curve includes 58 patients with samples exclusively from cluster 2, and the blue dotted curve includes 17 patients with samples from both cluster 1 and cluster 2. Patients with one or more samples from cluster 1 (red and blue curves) appear to have a similar prognosis, while patients with samples only from cluster 2 (black curve) appear to have significantly better prognosis

Combining sample-level information across tumor foci, we can categorize patients into three groups. The first group is comprised of patients all of whose samples are in cluster 1. The second group is made up of patients all of whose samples are in cluster 2. The third group consists of patients with samples in both cluster 1 and cluster 2 (labeled as *Mixed*). Additional file 1: Table S2 shows the distribution of samples between clusters stratified by the number of samples per patient. Kaplan-Meier survival curves for each of these three patient groups (Fig. 3) show that recurrence-free survival time is strongly differentiated by membership in one of the three groups (*p*-value = 1.3x10^−5^). A Cox proportional hazards model shows a statistically significant difference between the poor prognosis and good prognosis groups but no discernable difference between the poor prognosis group and the mixed group (Additional file 1: Table S3). The hazard ratios from a multivariate Cox regression (Additional file 1: Table S4) and Kaplan-Meier curves stratified by batch (Additional file 1: Figure S3) demonstrate that the results do not appear to be strongly dependent on the batch variable. While there appears to be a strong association between group membership and recurrence-free survival, it is useful to look at the relationship between these groups and current clinical criteria for transplantation.

### Association of microRNA expression with clinical covariates

Additional file 1: Figure S4 shows the association between the two microRNA-based subgroups and four clinical criteria for transplantation: (A) vascularization, (B) focality, and (C) number of tumors, as well as (D) the Milan criteria (a combination of the other criteria). In general, criteria that favor transplantation are less common in the poor prognosis subgroup (cluster 1). Of note, 100 % (50/50) of samples in the poor prognosis subgroup are from vascularized tumors, while only 24 % (30/126) of the samples in the good prognosis subgroup show increased vascularization. Also, 84 % (42/50) of samples in the poor prognosis subgroup are from patients who fall outside the Milan criteria. In contrast, 39 % (49/126) of samples in the good prognosis subgroup are from patients who fall within the Milan criteria. The Milan criteria reflect primarily patient-level measurements, so interpretation of their association with sample-level microRNA expression profiles is complicated by patients with samples in both subgroups. Additional file 1: Table S5 shows the distribution of patients across the Milan criteria and microRNA expression clusters. For the poor prognosis group, half (7/14) of the patients are outside Milan and half (7/14) are within Milan. For the good prognosis group the split is 30 of 58 within Milan and 28 of 58 outside Milan. The 17 patients in the mixed group are all outside Milan.

**Fig. 4:**
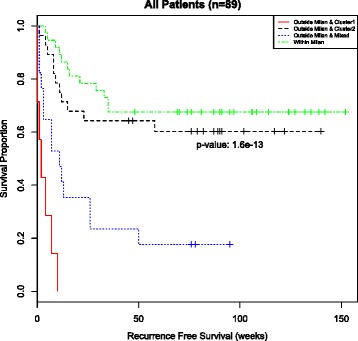
Kaplan-Meier curves of recurrence-free survival as delineated by Milan criteria and clustering on all 89 patients. The patients within Milan are not stratified by their microRNA expression profile because microRNA expression is not significantly associated with survival for patients within Milan (Figure S5). Recurrence-free survival time is strongly differentiated by subgroups defined by the Milan criteria and microRNA expression profiles (*p*-value = 1.6x10^−13^). Of note is that patients outside Milan whose samples all fall in cluster 2 (dashed black) have recurrence survival times that are nearly as good as patients within Milan (*p*-value = 0.44). Moreover, both of these subgroups have significantly different recurrence-free survival times than the other two subgroups: Outside Milan & Cluster 1 (7 patients) vs. Outside Milan & Cluster 2 (28 patients): *p*-value = 1.05x10^−7^; Outside Milan & Cluster 1 vs. Within Milan (37 patients): *p*-value = 1.03x10^−12^; Outside Milan & Mixed (17 patients) vs. Outside Milan & Cluster 2: *p*-value = 2.28x10^−3^; Outside Milan & Mixed vs. Within Milan: *p*-value = 1.89x10^−5^

Previous work reported a biomarker that was strongly associated with recurrence-free survival time independent of Milan status [6]. While our unsupervised sample-level analysis showed a strong association with recurrence-free survival (Fig. 3) and a similar association in patients outside Milan (Additional file 1: Figure S5A), it did not show a significant association with recurrence-free survival time for patients within Milan (Additional file 1: Figure S5B). In fact, patients outside Milan with microRNA expression profiles all in the good prognosis subgroup had similar recurrence-free survival to patients within Milan (Fig. 4), and both of these groups differed significantly from patients outside Milan with at least one poor prognosis sample. A Cox proportional hazards model shows a statistically significant difference between the Within Milan group and the Outside Milan & Cluster 1 (poor prognosis) group as well as the Outside Milan & Mixed group; however there was no discernable difference between the Within Milan group and the Outside Milan & Cluster 2 (good prognosis) group (Additional file 1: Table S6).

This latter group, Outside Milan & Cluster 2 (good prognosis), represents a group of patients who would be deemed unfit for liver transplantation based on the Milan criteria but would appear to have a good chance of recurrence free survival if they received a liver transplant. In summary, it appears that there is a subset of patients who would benefit from microRNA profiling when used with the Milan criteria to determine transplantation. However, it is unlikely that all 847 microRNAs used to differentiate between the good and poor prognosis subgroups are associated with HCC recurrence, nor would it be practical to perform microRNA profiling of tumor biopsies unless absolutely necessary. To obtain a clinically applicable microRNA biomarker, it is desirable to identify a subset of microRNAs that are actually associated with HCC recurrence.

### Feature selection

The previous results are based on the expression of 847 microRNAs in each sample. We hypothesize that a relatively small subset of these are the primary biomarkers for HCC recurrence. To find such features, we used mutual information as described in the Materials and Methods Section. After calculating the mutual information for each microRNA, we selected the top five features, which had mutual information values much greater than the rest (Additional file 1: Table S7). These five features are miR-122_st, miR-126_st, miR-15a_st, miR-22_st and miR-30a_st. Classification of patients into poor, mixed, and good prognosis clusters based on the expression of these five microRNAs exactly matched the classification using all 847 microRNAs. This means that the results shown in the Kaplan-Meier survival curves (Fig. 4) and Cox proportional hazard model (Additional file 1: Table S6) are identical whether one uses all 847 microRNAs or just these 5 microRNAs.

The first feature, miR-122, is specific to the liver and a reduced level of miR-122 is associated with HCC progression and metastasis [21]. Next, miR-126 regulates angiogenesis and is normally expressed in endothelial cells, such as capillaries and larger blood vessels. It is also associated with innate immune response [22]. Functioning as a tumor suppressor, miR-15a targets oncogene BCL2, and within tumor cells miR-15a itself is down regulated [23]. In several cancers, miR-22 has been shown to be associated with differentiation, metastasis and prognosis. In HCC, miR-22 is especially down regulated [24]. Down-regulation of miR-30a in HCC is strongly associated with decreased disease-free survival. In addition, tumor cell migration, invasion and epithelial-mesenchymal transition are associated with its down-regulation [25]. In a recent study on microRNA profiling in HCC vascular invasion, miR-122, miR-126, miR-15a, and miR-30a are down regulated in HCC samples with vascular invasion [26].

### Discretized microRNA expression is associated with recurrence-free survival

**Fig. 5:**
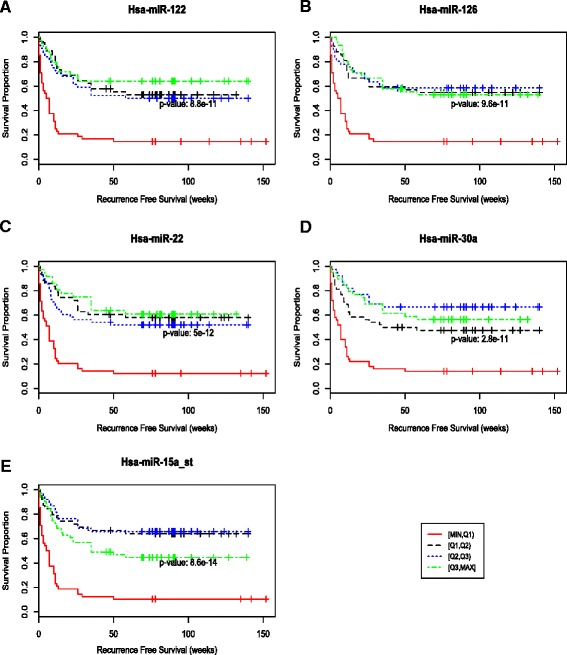
The relationship between microRNA expression and recurrence free survival time was examined for the five microRNAs with the highest mutual information: (**a**) miR-122_st, (**b**) miR-126_st, (**c**) miR-30a_st, (**d**) miR-22_st, and (**e**) miR-15a_st. Kaplan-Meier HCC recurrence free survival for 5 microRNAs. Each of the 4 curves in the figures indicates survival times for a different quartile of microRNA expression values, with red being the lowest values and green the highest. It is clear from the red curves representing the lowest quartile expression values, that poor recurrence-free survival is associated with low levels of expression of these 5 microRNAs—*p*-values: 8.8x10^−11^ (miR-122), 9.6x 10^−11^ (miR-126), 5X10^−12^ (miR-22), 2.8x10^−11^ (miR-30a), 8.6x10^−14^ (miR-15a)

Figure 5 shows the recurrence free survival curves based on the four different expression intervals for the top five microRNAs. Patients with samples in the lowest interval, [min,Q1], have significantly worse survival than those with samples in the other three intervals. This suggests an association between recurrence and reduced expression of these five microRNAs. Additional file 1: Figures S6–S10 show the expression values for the 5 miRNAs stratified by patient and colored by prognosis group. The centers shown in the figures (poor prognosis center and good prognosis center) are K-Means centers for each group.

The expression of these 5 microRNAs can be used to predict recurrence more expediently than using the full set of 847 microRNAs. To confirm the reduced feature set, we used supervised machine learning models to classify samples as poor or good prognosis. Specifically, we used naïve Bayes and support vector machine learning models with these five microRNA features. As described in the Material and Methods Section, half the samples were used for training and the other half for testing. The naïve Bayes model successfully classifies 28 of the testing samples as poor prognosis, and 60 as good prognosis. The support vector machine model replicates the naïve Bayes results. Given that the poor prognosis class was shown to be strongly associated with recurrence, we can conclude that these five microRNAs are an effective reduced feature set.

Finally, we examined the association between these five microRNAs and the measured clinical covariates. These 5 microRNAs appear to be associated with recurrence and vascularization, and to a lesser degree tumor stage, HCV, and the Milan criteria (Additional file 1: Figures S11–S15).

### Relationship to Previously Published Biomarker Approach

A previous approach to addressing within-subject heterogeneity in a subset of these data was to summarize from sample-level microRNA expression to patient-level profiles by considering the two extrema for each microRNA (the minimum and maximum observed values within each patient) [6]. This approach was used to identify a biomarker of HCC recurrence in a subset of the data considered in this manuscript. Of the five microRNAs identified in this manuscript, two were also part of the previous biomarker, miR-122 and miR-22.

To examine the performance of the previous approach, we applied the min/max procedure to summarize microRNA expression to the patient level. Here we consider three different min/max feature sets: (1) the 60 features with an FDR < 0.2 (all microRNAs in Table 2 of [6]), (2) the 6 features that were most consistently selected during cross-validation (bold microRNAs in Table 2 of [6]), and (3) 16 microRNAs previously reported in the literature as associated with HCC malignancy. We performed KMeans clustering on all 89 patients using each of these feature sets. Note that because the min/max procedure summarizes expression at the patient-level, clustering can be performed on patients rather than samples. While this differs from the biomarker methodology actually used in the original manuscript, it allows the most direct comparison to the results reported in this manuscript. MA-plots of the patient-level min/max expression profiles show a similar separation between recurrent and non-recurrent patients as the sample-level analysis (Additional file 1: Figures S16, S18, and S20). The 60 features seem to show the most separation between the two clusters; both the 16 and 6 feature sets result in a few samples that are approximately equidistant from both clusters.

Next, we examined the Kaplan-Meier recurrence-free survival curves based on each of the three feature sets (Additional file 1: Figures S17, S19, S21). All three feature sets were able to classify patients into good and poor prognosis groups (*p*-values < 0.0001). Finally, we discretized microRNA expression into the four ranges used for the five microRNA biomarker reported here and examined the associated between each of the six microRNAs in feature set 2 and recurrence-free survival (Additional file 1: Figure S22).

Finally, we fit a Cox proportional hazards model using each of the three min/max feature sets together with the Milan criteria to group patients. All three feature sets were able to distinguish between the Within Milan group and the Outside Milan & Cluster 1 (poor prognosis) group, and there was no discernable difference between the Within Milan group and the Outside Milan & Cluster 2 (good prognosis) group (Additional file 1: Tables S8-S9). However, the estimated hazard ratios were substantially less using the min/max feature sets. The 16 and 60 min/max feature sets produced identical results with hazard ratios of 5.91 with a 95 % confidence interval of (2.87, 12.16). The 6 min/max feature set resulted in a hazard ratio of 5.29 with a 95 % confidence interval of (2.58, 10.88). In contrast the 5 microRNA biomarker proposed in this manuscript yielded a hazard ratio of 18.93 with a 95 % confidence interval of (6.74, 53.14).

## Discussion

There are limited donor liver organs available for the HCC patients in need of liver transplantation. The discovery of biomarkers to predict HCC recurrence after liver transplantation is therefore important to appropriately use valuable organs. MicroRNAs are popular markers as they are logistically easy to obtain and can be effective in classifying tissue types and tumor tissues of origin [27]. Recent research has shown that a microRNA biomarker of HCC recurrence when used with the Milan criteria can improve prediction of recurrence post-transplantation [6].

HCC patients often present with multiple distinct tumor foci. The microRNA expression profiles from different samples in the same patient can differ significantly. This poses a challenge regarding which microRNA expression profiles from the same patient are most strongly associated with HCC recurrence. We assume that if HCC recurs after liver transplantation, there is at least one sample, and corresponding microRNA expression profile, that is responsible for the recurrence. For patients with multifocal disease this implies that not all foci are equally responsible for recurrence. Previous approaches either analyzed only one sample per patient [9–12] or used summarized sample-level information from multifocal patients [6], whereas our approach uses both sample-level and patient-level information to predict recurrence. This has implications for patients with highly heterogeneous microRNA expression profiles.

We identified five microRNAs that appear to be strongly associated with recurrence post transplantation. These five microRNAs (miR-122_st, miR-126_st, miR-15a_st, miR-22_st and miR-30a_st) are down regulated in samples from recurrent patients. This is consistent with previous research that reported down-regulation of miR-30a through comparisons of tumor and non-tumor tissue and showed reduced disease-free survival times are significantly associated with down regulation of miR-30a [25]. Both miR-126 and miR-122 have been previously reported as down regulated in HCC [11]. Finally, miR-15a was previously reported to be associated with shorter recurrence-free survival in HCC patients [9]. These five microRNAs represent a potential biomarker to predict HCC recurrence after liver transplantation, when used in concert with the Milan criteria. Development and validation of an assay to exclusively measure these five microRNAs is necessary to determine the clinical utility of the proposed biomarker.

The number of distinct tumor foci is associated with disease severity as well as recurrence post-transplantation, and together with tumor size, tumor number is a key component of the Milan criteria. Furthermore, patients for whom multiple foci were analyzed are more likely to exhibit heterogeneous microRNA expression profiles simply due to a greater number of samples undergoing genomic analysis. For this reason, we do not assign special significance to the mixed subgroup (Figs. 3 and 4), rather we consider this subgroup to be comparable to the poor prognosis subgroup. Patients in either of these subgroups have at least one poor prognosis sample and have generally poorer recurrence free survival following transplantation. However, several studies have reported intra-tumor heterogeneity itself as a predictor of prognosis [28, 29] and suggested focusing further investigations on the underlying causes of heterogeneity [30].

A key finding is that for patients with heterogeneous miRNA expression values, a subset of the within-patient values can be statistically significant predictors of post transplant recurrence. In particular, there are 17 patients from the mixed group who have samples from both clusters (Additional file 1: Table S2). Patient-level summaries for these patients based on average expression are potentially misleading due to the heterogeneity between samples from the same patient. Failure to account for within-patient heterogeneity can negatively impact both biomarker development and application. Furthermore, patients with heterogeneous microRNA expression profiles consistently fall outside the Milan criteria (Additional file 1: Table S5) and have recurrence free survival comparable to patients with purely poor prognosis microRNA expression profiles (Fig. 3).

In this work, we have focused on within-patient tumor heterogeneity. Heterogeneity has also been reported within individual samples [31, 32] and between patients with the same cancer subtype [33]. While methods have been developed to address each of these types of heterogeneity [34, 35], careful modeling of these sources of heterogeneity remains an important challenge in genomic medicine.

Despite these limitations, the results reported in this manuscript suggest that microRNA expression profiling of distinct tumor foci could improve prediction of recurrence and therefore aid in determining candidates for transplantation. Specifically, measuring the expression of these five microRNAs may represent a low cost addition to standard evaluation. However, increased data collection comes with a cost – very small tumors with definitive radiographic characteristics of HCC are seen frequently but difficult to biopsy because of their small size. Furthermore, small nodules can be difficult to distinguish from regenerative nodules in the cirrhotic liver. Finally, one must weigh the additional information gained against the risk of multiple biopsies; in HCC, one is often biopsying a diseased liver with an increased risk of bleeding. When biopsy is combined with a tumor ablative technique such as radiofrequency ablation, bleeding risks are minimized by cauterizing along the biopsy needle tract. Therefore, tumor sampling for genomic analysis is perhaps best performed in the Interventional Radiology Suite or the operating room. The clinical challenge in surveying the entire tumor burden in a patient is formidable and will require a combination of advances in both medical and genomic techniques.

## Conclusions

In this paper we propose a new approach to combine sample-level and patient-level information to discover microRNA biomarkers of HCC recurrence after liver transplantation. Five specific microRNAs are suggested as a putative biomarker. It appears that there is a subset of patients who would benefit from microRNA profiling when used with the Milan criteria to determine transplantation.

### Ethics approval and consent to participate

This study was performed with approval of the University of Rochester Research Subjects Review Board (RSRB00029467). Liver explant pathology specimens (paraffin embedded blocks) from patients undergoing liver transplant for HCC were de-identified prior to processing and analysis, so individuals were exempt from consent.

### Consent to publish

Not applicable.

### Availability of data and materials

Raw data are available via the Gene Expression Omnibus (GEO) with accession number GSE67140: http://www.ncbi.nlm.nih.gov/geo/query/acc.cgi?acc=GSE67140. Processed and annotated data, as well as R scripts for the analyses are freely available on GitHub: https://github.com/mccallm/HCCmicroRNA.

### Description of additional data files

The Supplementary Materials contain additional Figures (S1–S22) and Tables (S1–S9) referenced in the manuscript. All supplementary tables and figures are included in a single file: SupplementaryMaterials.docx.

## Additional file

Additional file 1: Supplementary Materials. (DOCX 3656 kb)

## Abbreviations

GEO: Gene Expression Omnibus
HCC: Hepatocellular carcinoma
PCA: Principal components analysis
t-SNE: t-Distributed stochastic neighbor embedding
