# Supplementary material for: A microRNA biomarker of hepatocellular carcinoma recurrence following liver transplantation accounting for within-patient heterogeneity
Source: BMC Med Genomics. 2016 Apr 8;9:18. doi: 10.1186/s12920-016-0179-4 (PMC4826548; doi:10.1186/s12920-016-0179-4)
Supplement: Supplementary file 1 — Supplementary Materials. (DOCX 3656 kb) [file 12920_2016_179_MOESM1_ESM.docx]

# Support Information

**Table S1** Patient cohort demographics (total population=89)

|  |  | No Recurrence(n=45) | Recurrence (n=44) |
| --- | --- | --- | --- |
| Focality | Biifocal | 1 |  |
|  | Multifocal | 29 | 32 |
|  | Unifocal | 15 | 12 |
| Number of tumors | 1 | 15 | 12 |
|  | 2 | 9 | 3 |
|  | 3 | 12 | 10 |
|  | 4 | 1 | 4 |
|  | 5 | 1 |  |
|  | Multiple | 7 | 15 |
| Milan Criteria | Outside | 20 | 32 |
|  | Within | 25 | 12 |
| HCV | Neg | 21 | 22 |
|  | Pos | 24 | 22 |
| Tumor Grade | Moderately Differentiated | 13 | 22 |
|  | Moderately-Poorly Differentiated | 1 | 4 |
|  | Poorly Differentiated | 1 | 5 |
|  | Well differentiated | 23 | 10 |
|  | Well-moderately differentiated | 7 | 3 |
| Tumor Stage | I | 11 | 6 |
|  | II | 25 | 12 |
|  | IIIA | 8 | 25 |
|  | IIIB | 1 | 1 |
| Vascular Invasion | N | 34 | 13 |
|  | Y | 11 | 31 |
| Largest(Mean) Tumor Size (cm) |  | 3.48 | 5.74 |
| Etiology | A1A | 1 | 0 |
|  | AIH | 1 | 0 |
|  | Cryptogenic | 5 | 6 |
|  | HBV | 4 | 4 |
|  | HCV | 23 | 21 |
|  | Hemochromatosis | 2 | 1 |
|  | Laennec's | 5 | 5 |
|  | NASH | 4 | 6 |
|  | Primary HCC | 0 | 1 |

**Table S2** Patient and per cluster sample counts

|  | 🡨 Cluster 1 Cluster 2 🡪 | | | | | | | | | | Total |
| --- | --- | --- | --- | --- | --- | --- | --- | --- | --- | --- | --- |
| Patients with 1 sample | 8 (1,0) | | | | | 27 (0,1) | | | | | 35 |
| Patients with 2 samples | 1 (2,0) | | | 5 (1,1) | | | | 20 (0,2) | | | 26 |
| Patients with 3 samples | 4 (3,0) | | 2 (2,1) | | | 6 (1,2) | | | 11 (0,3) | | 23 |
| Patients with 4 samples | 1 (4,0) | 2 (3,1) | | | 1 (2,2) | | 1 (1,3) | | | 0 (0,4) | 5 |
|  |  |  | | |  | |  | | |  | 89 |

Note: In each cell, the number of patients and the number of samples in each cluster are shown. For example, 6 (1,2), means 6 patients which have 1 sample from cluster 1 and 2 samples from cluster 2. 2 (3,1) means that 2 patients have 3 samples in cluster 1 and 1 sample in cluster 2.

**Table S3** Cox proportional hazard model corresponding to figure 3

| **Cluster** | **Coef:β** | **HR:exp(β)** | **95%CI** | **ρ-value** |
| --- | --- | --- | --- | --- |
| 1 (Poor prognosis) | Baseline Reference | | | |
| 2 (Good Prognosis) | -1.28 | 0.28 | [0.13 0.60] | 0.001 |
| 3 (Mixed) | 0.11 | 1.11 | [0.49 2.51] | 0.80 |

Note: HR=hazard ratio, CI=confidence interval.

**Table S4**  Hazard ratios from multivariate Cox PH

|  | **Coef:β** | **HR:exp(β)** | **95% CI** | **ρ-value** |
| --- | --- | --- | --- | --- |
| **Cluster** | | | | |
| 1 (Poor prognosis) | Baseline Reference | | | |
| 2 (Good prognosis) | -1.49 | 0.23 | [0.14 0.36] | 7.58E-10 |
| **Batch group** | | | | |
| 1 | Baseline Reference | | | |
| 2 | 0.25 | 1.28 | [0.73 2.25] | 0.39 |
| 3 | 0.13 | 1.14 | [0.55 2.35] | 0.73 |
| 4 | -0.34 | 0.71 | [0.42 1.22] | 0.22 |

Note: HR=hazard ratio, CI=confidence interval.

**Table S5**  Patient count per cluster, within and outside milan criteria

|  | **Cluster 1** (Poor Prognosis) | | **Cluster 2**  (Good Prognosis) | | **Cluster 3** (Mixed Group) | |
| --- | --- | --- | --- | --- | --- | --- |
| Milan Criteria | Within | Outside | Within | Outside | Within | Outside |
| Patients with 1 sample | 6 | 2 | 18 | 9 | 0 | 0 |
| Patients with 2 samples | 1 | 0 | 5 | 15 | 0 | 5 |
| Patients with 3 samples | 0 | 4 | 7 | 4 | 0 | 8 |
| Patients with 4 samples | 0 | 1 | 0 | 0 | 0 | 4 |

**Table S6** Cox proportional hazard model corresponding to Figure 4

| **Cluster & Milan** | **Coef:β** | **HR:exp(β)** | **95%CI** | **ρ-value** |
| --- | --- | --- | --- | --- |
| Within Milan | Baseline Reference | | | |
| Outside Milan & Cluster 1 (poor prognosis) | 2.94 | 18.93 | [6.74 53.14] | 2.34E-08 |
| Outside Milan & Cluster 2 (good prognosis) | 0.31 | 1.37 | [0.60 3.10] | 0.46 |
| Outside Milan & Mixed | 1.53 | 4.64 | [2.12 10.10] | 0.0001 |

Note: HR=hazard ratio, CI=confidence interval.

**Table S7** Mutual information values

|  | **miRNA** | **Mutual Information** | **Interval** | **Max Value for Interval** |
| --- | --- | --- | --- | --- |
| 1 | hsa-miR-122_st | 0.811278124 | m1 | 12.96621286 |
| 2 | hsa-miR-126_st | 0.811278124 | m1 | 8.156695285 |
| 3 | hsa-miR-15a_st | 0.811278124 | m1 | 3.887445887 |
| 4 | hsa-miR-22_st | 0.811278124 | m1 | 9.82923286 |
| 5 | hsa-miR-30a_st | 0.811278124 | m1 | 5.764202823 |
| 6 | hsa-let-7g_st | 0.65963115 | m1 | 5.762968311 |
| 7 | hsa-miR-100_st | 0.65963115 | m1 | 7.281452659 |
| 8 | hsa-miR-106b_st | 0.65963115 | m1 | 7.306054194 |
| 9 | hsa-miR-1307_st | 0.65963115 | m4 | 10.16597294 |
| 10 | hsa-miR-146b-5p_st | 0.65963115 | m1 | 4.591633582 |
| 11 | hsa-miR-148a_st | 0.65963115 | m1 | 2.648607237 |
| 12 | hsa-miR-152_st | 0.65963115 | m1 | 4.125526393 |
| 13 | hsa-miR-16_st | 0.65963115 | m1 | 9.910046327 |
| 14 | hsa-miR-195_st | 0.65963115 | m1 | 5.144519875 |
| 15 | hsa-miR-21_st | 0.65963115 | m1 | 3.250119013 |
| 16 | hsa-miR-28-5p_st | 0.65963115 | m1 | 4.057902933 |
| 17 | hsa-miR-29a_st | 0.65963115 | m1 | 7.677781717 |
| 18 | hsa-miR-30e_st | 0.65963115 | m1 | 3.233985451 |
| 19 | hsa-miR-99a_st | 0.65963115 | m1 | 8.789886043 |
| 20 | hsa-miR-10a_st | 0.554471925 | m1 | 2.502024535 |
| 21 | hsa-miR-1281_st | 0.554471925 | m4 | 10.65194543 |
| 22 | hsa-miR-151-3p_st | 0.554471925 | m1 | 6.037315626 |
| 23 | hsa-miR-185_st | 0.554471925 | m1 | 7.705051487 |
| 24 | hsa-miR-192_st | 0.554471925 | m1 | 8.915323897 |
| 25 | hsa-miR-194_st | 0.554471925 | m1 | 9.669802157 |
| 26 | hsa-miR-20b_st | 0.554471925 | m1 | 4.362298645 |
| 27 | hsa-miR-26a_st | 0.554471925 | m1 | 11.37338516 |
| 28 | hsa-miR-30a-star_st | 0.554471925 | m1 | 2.597794941 |
| 29 | hsa-miR-455-5p_st | 0.554471925 | m1 | 2.282988247 |
| 30 | hsa-miR-494_st | 0.554471925 | m4 | 12.05278997 |
| 31 | hsa-let-7f_st | 0.467545459 | m1 | 5.345997543 |
| 32 | hsa-let-7i_st | 0.467545459 | m1 | 8.416166773 |
| 33 | hsa-miR-1207-5p_st | 0.467545459 | m4 | 12.21488474 |
| 34 | hsa-miR-149-star_st | 0.467545459 | m4 | 13.42573882 |
| 35 | hsa-miR-155_st | 0.467545459 | m1 | 5.919080438 |
| 36 | hsa-miR-19b_st | 0.467545459 | m1 | 5.825981806 |
| 37 | hsa-miR-20a_st | 0.467545459 | m1 | 8.340439056 |
| 38 | hsa-miR-30d_st | 0.467545459 | m1 | 7.462537776 |
| 39 | hsa-miR-335_st | 0.467545459 | m4 | 9.028396195 |
| 40 | hsa-miR-34a_st | 0.467545459 | m1 | 8.565358951 |
| 41 | hsa-miR-768-5p_st | 0.467545459 | m4 | 11.98392655 |
| 42 | hsa-miR-933_st | 0.467545459 | m4 | 9.923363124 |
| 43 | hsa-miR-106a_st | 0.392883963 | m1 | 8.954178571 |
| 44 | hsa-miR-1225-5p_st | 0.392883963 | m4 | 11.19978545 |
| 45 | hsa-miR-1268_st | 0.392883963 | m4 | 11.96075869 |
| 46 | hsa-miR-1275_st | 0.392883963 | m4 | 10.69944879 |
| 47 | hsa-miR-128_st | 0.392883963 | m1 | 2.503251583 |
| 48 | hsa-miR-143_st | 0.392883963 | m1 | 8.638342811 |
| 49 | hsa-miR-146a_st | 0.392883963 | m1 | 3.755565535 |
| 50 | hsa-miR-17_st | 0.392883963 | m1 | 9.025288414 |
| 51 | hsa-miR-193a-3p_st | 0.392883963 | m1 | 3.73152119 |
| 52 | hsa-miR-199b-3p_st | 0.392883963 | m1 | 5.295659949 |
| 53 | hsa-miR-25_st | 0.392883963 | m1 | 6.716569777 |
| 54 | hsa-miR-27a_st | 0.392883963 | m1 | 7.304212873 |
| 55 | hsa-miR-28-3p_st | 0.392883963 | m1 | 5.458183305 |
| 56 | hsa-miR-29b_st | 0.392883963 | m1 | 2.237384428 |
| 57 | hsa-miR-30b_st | 0.392883963 | m1 | 6.306025582 |
| 58 | hsa-miR-339-3p_st | 0.392883963 | m1 | 5.146812014 |
| 59 | hsa-miR-744_st | 0.392883963 | m4 | 10.00965291 |
| 60 | hsa-miR-92b-star_st | 0.392883963 | m4 | 10.9342141 |
| 61 | hsa-miR-940_st | 0.392883963 | m4 | 9.19865533 |

Note: Values for each miRNA feature are binned into four intervals, [Min,Q1), [Q1,Q2), [Q2,Q3), [Q3,Max]. They are indicated by m1, m2, m3, m4 respectively. Here Q1, Q2, Q3 are the first, second, third quartiles for each feature’s values. The Max Value for Interval column shows the highest expression value of samples in the interval indicated for the given feature.

**Table S8** Cox proportional hazard model using either the 60 or 16 min/max feature set

| **Cluster & Milan** | **Coef:β** | **HR:exp(β)** | **95%CI** | **ρ-value** |
| --- | --- | --- | --- | --- |
| Within Milan | Baseline Reference | | | |
| Outside Milan & Cluster 1 (poor prognosis) | 1.78 | 5.91 | [2.87 12.16] | 1.42E-06 |
| Outside Milan & Cluster 2 (good prognosis) | 0.30 | 1.36 | [0.60 3.07] | 0.47 |

**Table S9** Cox proportional hazard model using the 6 min/max feature set

| **Cluster & Milan** | **Coef:β** | **HR:exp(β)** | **95%CI** | **ρ-value** |
| --- | --- | --- | --- | --- |
| Within Milan | Baseline Reference | | | |
| Outside Milan & Cluster 1 (poor prognosis) | 1.67 | 5.29 | [2.58 10.88] | 5.49E-06 |
| Outside Milan & Cluster 2 (good prognosis) | 0.35 | 1.42 | [0.63 3.22] | 0.40 |

**Figure S1**

176 samples with 847 dimensions are mapped into two dimensions via Principal Components Analysis (PCA). Samples closer together have more similar microRNA expression profiles. The samples from HCC recurrent patients are indicated with red triangles. The samples from non-recurrent patients are indicated with black circles. The majority of samples on the right are from recurrent patients; those on the left are from a mixture of recurrent and non-recurrent patients.

**Figure S2** MA-plot of distance between the miRNA expression profile of each sample and the average expression profile of each subgroup. Axis interpretation: Difference in distance to cluster centers (M=d2-d1), Average distance to cluster centers (A=(d1+d2)/2). Large values of M correspond to samples that are far more similar to the average expression profile of subgroup 1 than subgroup 2. Large values of A correspond to samples that differ from the average expression profile of both subgroups.

**Figure S3** Kaplan-Meier curves of recurrent-free survival for four batch groups. The red cluster 1 curve includes patients with samples from cluster 1 only. The black dashed cluster 2 curve includes patients with samples exclusively from cluster 2, and the blue dotted mixed curve includes patients with samples from both cluster 1 and cluster 2.

**Figure S4** Samples distribution association with four clinical covariates. Axis interpretation: Difference in distance to cluster centers (M=d2-d1), Average distance to cluster centers (A=(d1+d2)/2). Large values of M correspond to samples that are far more similar to the average expression profile of subgroup 1 than subgroup 2. Large values of A correspond to samples that differ from the average expression profile of both subgroups.

**Figure S5** Kaplan-Meier curves of recurrence-free survival as delineated by KMeans clustering on patients. A: outside of Milan Criteria B: within Milan Criteria. The red cluster 1 curve includes patients with samples from cluster 1 only. The black dashed cluster 2 curve includes patients with samples exclusively from cluster 2, and the blue dotted mixed curve includes patients with samples from both cluster 1 and cluster 2. No patients from the mixed group were within Milan.

**Figure S6** miRNA-126 expression values and cluster membership for each sample from each patient.

**Figure S7** miRNA-122 expression values and cluster membership for each sample from each patient.

**Figure S8**  miRNA-22 expression values and cluster membership for each sample from each patient.

**Figure S9** miRNA-30a expression values and cluster membership for each sample from each patient.

**Figure S10** miRNA-15a expression values and cluster membership for each sample from each patient.

**Figure S11** Five miRNAs & HCC recurrence. The Y-axis shows absolute expression values for each miRNA sample for both recurrent (red) and non-recurrent (green) boxplots. It can be seen that the expression values are generally lower for recurrence.

**Figure S12** Five miRNAs & vascularization. The Y-axis shows absolute expression values for each miRNA sample for both vascularized (red) and non-vascularized (green) boxplots. It can be seen that the expression values are generally lower for vascularization.

**Figure S13** Five miRNAs & tumor stage. The Y-axis shows absolute expression values for each miRNA sample for tumor stage I, II, IIIA and IIIB boxplots. It can be seen that median expression values are generally lower for stages IIIA and IIIB vs. I and II.

**Figure S14** Five miRNAs & HCV. The Y-axis shows absolute expression values for each miRNA sample for both HCV (red) and non-HCV (green) boxplots. It can be seen that the median expression values are generally higher for HCV, but with many lower-valued outlier samples except for hsa-miR-15a.

**Figure S15** Five miRNAs & milan criteria. The Y-axis shows absolute expression values for each miRNA sample for both outside Milan (red) and within Milan (green) boxplots. It can be seen that the expression values are generally lower for outside Milan.

**Figure S16** The distribution of 89 patients with 60 Min-Max features. Axis interpretation: Difference in distance to cluster centers (M=d2-d1), Average distance to cluster centers (A=(d1+d2)/2). Large values of M correspond to samples that are far more similar to the average expression profile of subgroup 1 than subgroup 2. Large values of A correspond to samples that differ from the average expression profile of both subgroups. A black circle indicates a patient from cluster 1. A red triangle indicates a patient from cluster 2.

**Figure S17** Kaplan-Meier HCC recurrence free survival curves ( 89 patients with 60 min-max features). The black indicates patients from cluster 1. The red dashed curve indicates patients from cluster 2.


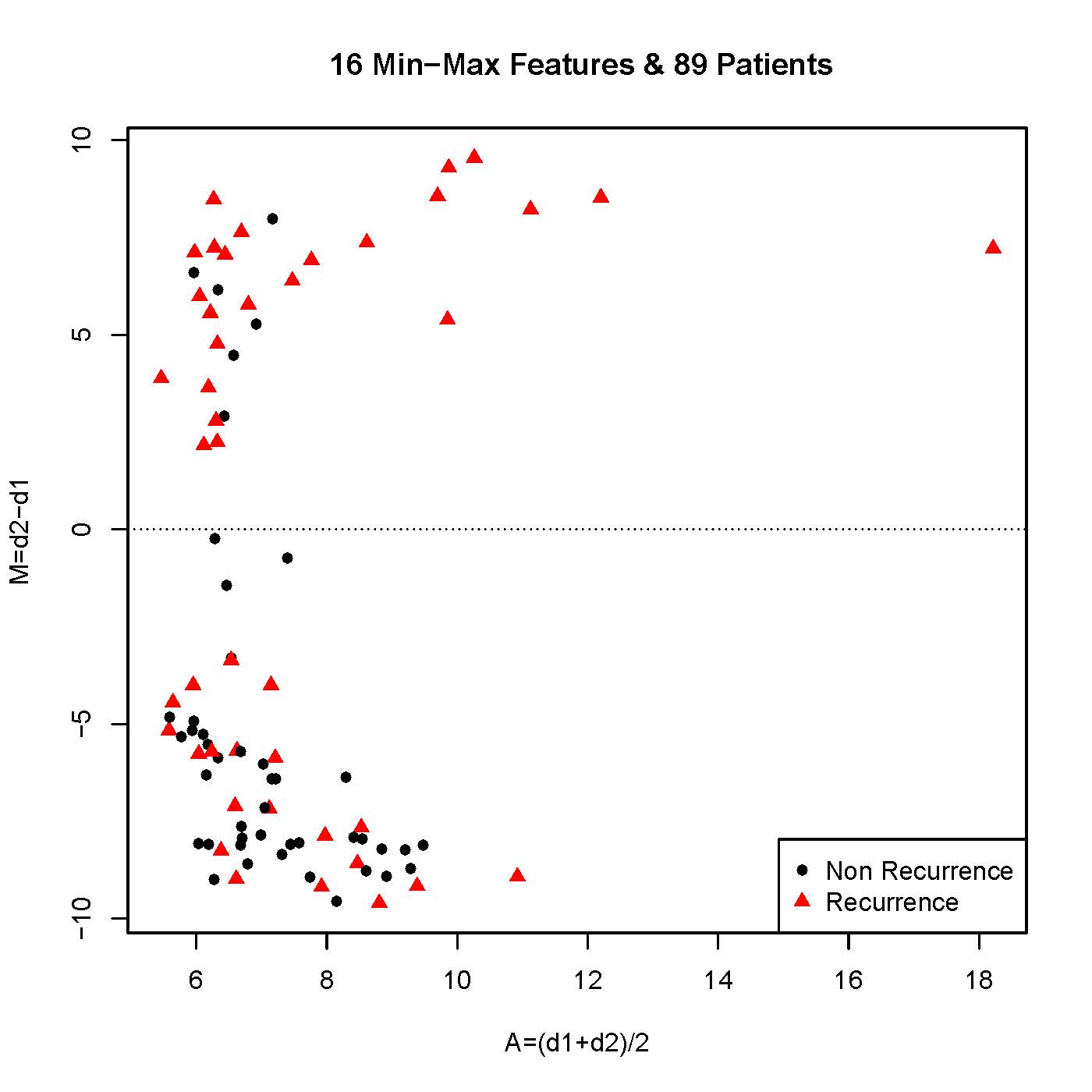


**Figure S18** Distribution of 89 Patients by 16 Min-Max features. Axis interpretation: Difference in distance to cluster centers (M=d2-d1), Average distance to cluster centers (A=(d1+d2)/2). Large values of M correspond to samples that are far more similar to the average expression profile of subgroup 1 than subgroup 2. Large values of A correspond to samples that differ from the average expression profile of both subgroups. A black circle indicates a patient from cluster 1. A red triangle indicates a patient from cluster 2.


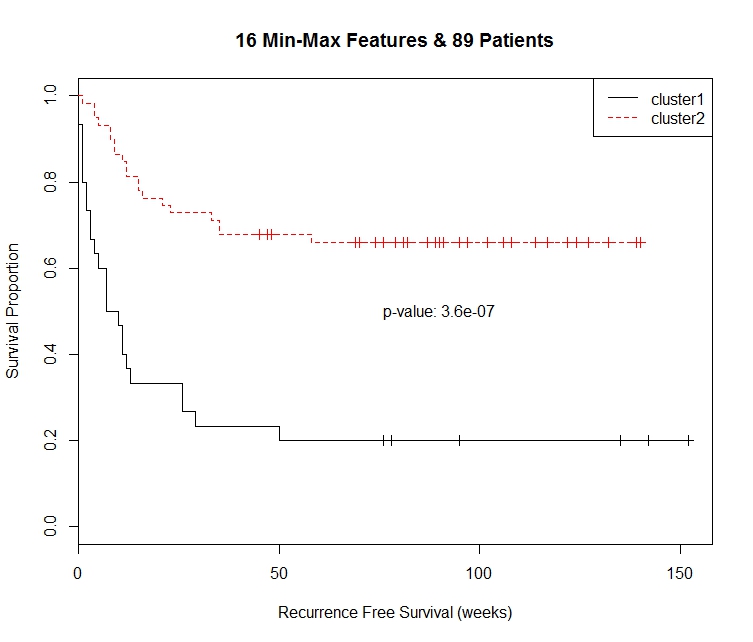


**Figure S19** Kaplan Meier recurrence-free survival curve (89 patients with 16 Min-Max features). The black indicates patients from cluster 1. The red dashed curve indicates patients from cluster 2.


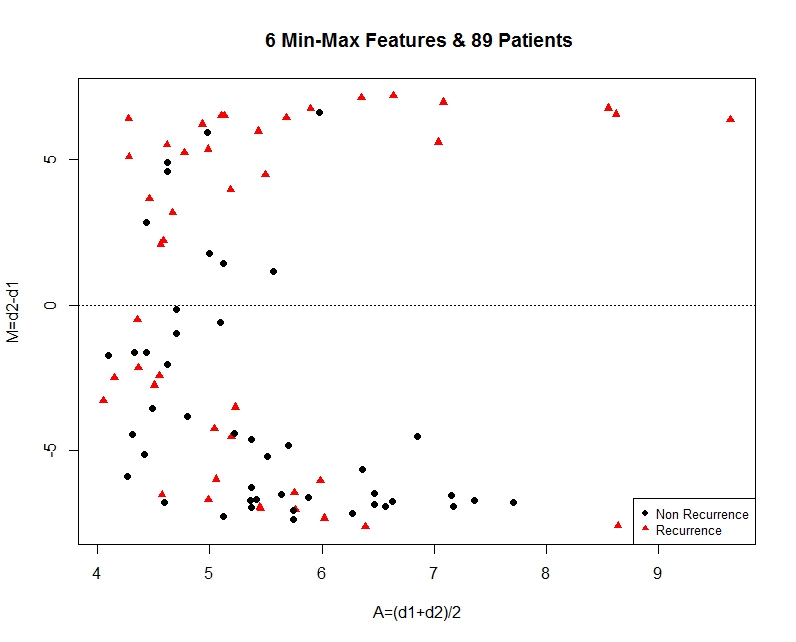


**Figure S20** Distribution of 89 patients by 6 Min-Max features. Axis interpretation: Difference in distance to cluster centers (M=d2-d1), Average distance to cluster centers (A=(d1+d2)/2).


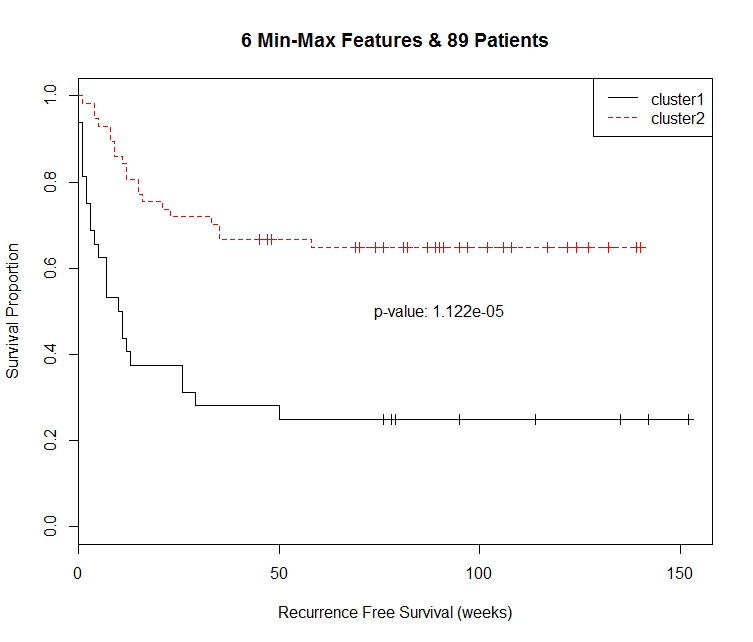


**Figure S21** Kaplan Meier recurrence-free survival curve (6 Min-Max features). The black indicates patients from cluster 1. The red dashed curve indicates patients from cluster 2.

**Figure S22** Kaplan Meier recurrence-free survival curve (6 Min-Max patient-level features) for value interval [Min, Q1], [Q1,Q2], [Q3,Max]. Here Q1, Q2, Q3 are the first, second, third quartiles of each feature.
